# Volatile Organic Metabolites as Potential Biomarkers for Genitourinary Cancers: Review of the Applications and Detection Methods

**DOI:** 10.3390/metabo15010037

**Published:** 2025-01-10

**Authors:** Kiana L. Holbrook, Wen-Yee Lee

**Affiliations:** Department of Chemistry and Biochemistry, University of Texas at El Paso, El Paso, TX 79968, USA; kholbrook@miners.utep.edu

**Keywords:** genitourinary cancer, volatile organic compounds, biomarkers, gas chromatography–mass spectrometry, liquid chromatography–mass spectrometry

## Abstract

Cancer is one of the leading causes of death globally, and is ranked second in the United States. Early detection is crucial for more effective treatment and a higher chance of survival rates, reducing burdens on individuals and societies. Genitourinary cancers, in particular, face significant challenges in early detection. Finding new and cost-effective diagnostic methods is of clinical need. Metabolomic-based approaches, notably volatile organic compound (VOC) analysis, have shown promise in detecting cancer. VOCs are small organic metabolites involved in biological processes and disease development. They can be detected in urine, breath, and blood samples, making them potential candidates for sensitive and non-invasive alternatives for early cancer detection. However, developing robust VOC detection methods remains a hurdle. This review outlines the current landscape of major genitourinary cancers (kidney, prostate, bladder, and testicular), including epidemiology, risk factors, and current diagnostic tools. Furthermore, it explores the applications of using VOCs as cancer biomarkers, various analytical techniques, and comparisons of extraction and detection methods across different biospecimens. The potential use of VOCs in detection, monitoring disease progression, and treatment responses in the field of genitourinary oncology is examined.

## 1. Introduction

Cancer is defined and characterized by the uncontrolled growth of abnormal cells that permeate, metastasize, and destroy normal cells. It remains a global health concern and has been estimated to be the second leading cause of mortality in the United States [1,2,3]. According to the American Cancer Society, it is estimated that, in 2024, roughly 2 million new cases will be diagnosed and that approximately 609,820 Americans will succumb to cancer [2]. Future estimations suggest that cancer death tolls will rise to over 13.1 million by the year 2030 [4]. With the projected increases in cancer mortality and risk, there is a dire need for early cancer detection and screening techniques to improve recovery, as well as the administration of appropriate treatment to increase the chance of survival [5]. Recent developments in detection and treatment have increased the overall cancer 5-year survival rates from roughly 50 percent in the 1970s to 69 percent and even 90 percent in some cancers during 2013–2019 [1,2,6]. Even though cancer diagnoses and detection methods have drastically evolved over the years, many of the tests and techniques present great invasiveness and often present limitations. These limitations and challenges include a high risk of false-positive interpretation, invasiveness, misdiagnosis, and over-diagnosis [7,8,9]. Thus, continuing research is needed to address those concerns.

### 1.1. Kidney Cancer

Kidney cancer is ranked the 6th most common cancer in men and the 9th most common in women. Men have almost twice the lifetime risk of receiving kidney cancer compared to their female counterparts, with the median age of diagnosis between 55 and 74 years of age [2]. The incidence and mortality rates of kidney cancer among all cancers in the United States for 2024 were estimated at 4% and 2%, respectively [2]. The most common type of kidney cancer is renal cell carcinoma (RCC), accounting for approximately 90% of kidney cancer [10]. RCC is a malignancy that originates in the tubule/cortex lining of the kidney, where the primary functionalities of these small tubules are to filter blood and produce urine. RCC is classified into three major malignancy subtypes based on morphology, genetics, and histology: clear cell renal cell carcinoma (ccRCC), papillary renal cell carcinoma (pRCC), chromophobe renal cell carcinoma (chRCC), and various rare and less commonly diagnosed subtypes, accounting for 75–85%, 10–15%, 5–10%, and 1–5%, respectively [10,11]. Compared to other cancers, kidney cancer shows great outcomes if detected in its early stages [12]. The variability in the survival rate has shown a high correlation to various factors attributed to the first diagnosis (i.e., stage, cancer morphology, location, age, sex, and other predisposed health risks) [2]. Generally, two-thirds of diagnoses are localized to one kidney, representing a 93% increase in the five-year survival rate [2,4]. Depending on the metastasis and aggressive nature of the tumor, when cancer begins to spread to proximal tissues, organs, or within the lymph nodes, survival decreases to 70%. Specifically, in metastatic kidney cancer, the survival rate drastically decreases to 12% [3], thus highlighting the clinical importance of early detection. Currently, there is no routine screening test for kidney cancer for people who are at average or high risk. Certain imaging tests, like ultrasound or MRI (Magnetic Resonance Imaging), could be used to detect the early signs of cancer, but these tests are expensive.

### 1.2. Prostate Cancer

Prostate cancer (PCa) is the second leading cause of cancer-related death among American men [3,13]. The proliferation of cancerous cells originates in the prostate gland, which functions primarily as the locale of seminal fluid creation. According to the Centers for Disease Control and Prevention (CDC) and American Cancer Society (ACS), 13 out of 100 American men will be diagnosed with prostate cancer during their life, with a 1 in 41 mortality rate. The highest incidence rates have been found in African American men above the age of 55 [3,14]. One of the major risk factors is attributed to genetic inheritance and age [15,16,17]. The recent literature has suggested that genetic disposition accounted for 5–10%, while 30–40% are commonly attributed to gene mutations [13]. In 2024, it was estimated that 299,010 new cases will be diagnosed in the United States alone and contribute to 35,250 deaths [2]. Early-stage prostate cancer may not produce noticeable symptoms, emphasizing the importance of regular screenings for monitoring and early detection. Non-invasive methods, such as prostate-specific antigen (PSA) tests and digital rectal exams (DRE), are generally used for PCa screening. These tests can be conducted in routine office visits without requiring specialized equipment or radiology expertise. However, PSA levels have been known to increase due to other factors unrelated to PCa, which in turn causes over-diagnosis and decrements in its accuracy [18,19,20,21,22]. All of these issues around PSA highlight the clinical need for alternative screening tools that can more accurately identify men who are most likely to benefit from early diagnosis and treatment while avoiding the over-diagnosis of clinically insignificant or low-grade PCa.

Multiparametric prostate MRI (mpMRI) is a useful tool in diagnosing, staging, and managing prostate cancer, combining T2-weighted, diffusion-weighted, and dynamic contrast-enhanced imaging to provide a detailed view of prostate anatomy and abnormalities. By improving the detection of clinically significant cancers, mpMRI helps distinguish aggressive tumors from indolent ones, reducing unnecessary biopsies and guiding targeted biopsies in areas of high suspicion. Like any technique, there are some drawbacks. mpMRI is costly and may have limited patient accessibility, has variable accuracy when patients have small or low-grade cancers, can be timely/uncomfortable, and uses contrast agents. As a comparison, the aforementioned non-invasive methods are generally quicker, more accessible, and less costly than mpMRI. Non-invasive methods are particularly useful as initial screening tools because they help identify individuals at higher risk who may then be referred for more in-depth imaging, like mpMRI, if needed. This tiered approach allows for efficient resource use, reserving mpMRI for cases where additional diagnostic precision is required to confirm or further evaluate a potential cancer diagnosis.

### 1.3. Bladder Cancer

Bladder cancer (BCa) is a disease in which abnormal cells grow uncontrollably in the bladder. The most common type is urothelial carcinoma, which starts in the urothelial cells lining the bladder. BCa is the tenth most common malignancy worldwide, accounting for a four times greater incidence in men than women and displaying a two times greater incidence in white men compared to other races (standardized by age and locality) [3,23]. According to the American Cancer Society, in 2024, there was projected to be 83,190 new cases, and projections suggested the result of 16,840 deaths [2]. Prior to 1991, the number of BCa cases rose and then declined by 29% over time to the past year, 2023 [24]. There are three main histological subtypes that are attributed to BCa: transitional cell carcinoma (TCC, 90%), and a combination of the rarer cases of squamous cell carcinoma (SCC) and adenocarcinoma/glandular type, accounting for 10%. Muscle invasive bladder cancer (MIBC) accounts for 20% of bladder cancer cases and has the highest mortality rate due to metastasis [3,24]. Some risk factors, including smoking, environmental factors, and chemicals of arsenic-contaminated drinking water, have been attributed to more than half of the incidences of this cancer [25]. Tobacco smoking has been correlated with increased metastasis frequency in bladder, pancreatic, and breast cancers [26,27]. Bladder cancer’s five-year survival rate is 77%, with 51% accounting for early diagnosis prior to metastasis, where the survival rate is 96% [3,28]. Similar to renal cancer, there are no standardized early detection methods for BCa [29,30]. The literature has shown promising biomarkers, but extensive research is needed to validate those biomarkers to be sensitive, specific, and reliable [28,31,32].

### 1.4. Testicular Cancer

Testicular cancer is a relatively rare form of cancer, accounting for about 1% of cancers in men, but it is the most common cancer in young men between the ages of 15 and 35. The American Cancer Society estimates that in the United States alone, around 9760 new cases of testicular cancer will be diagnosed each year, and about 500 men will die from the disease [2]. Testicular cancer has a notably high survival rate when caught early; the five-year survival rate for localized testicular cancer is about 99%, while the overall five-year survival rate, including cases where cancer has spread regionally, is around 95% [33,34,35]. These high survival rates are due to advancements in treatment, including surgery, radiation, and chemotherapy, as well as improvements in early detection. Most cases of testicular cancer originate in germ cells, which produce sperm and are categorized as either seminomas or non-seminomas. Seminomas tend to grow more slowly and respond well to radiation therapy, while non-seminomas are generally more aggressive but respond well to chemotherapy [36,37]. Risk factors for testicular cancer include a history of an undescended testicle, abnormal testicle development, family history, and certain genetic conditions. Although testicular cancer is highly treatable, regular self-examinations and awareness of symptoms, such as lumps or swelling in the testicles, are essential for early detection and successful treatment outcomes [38]. Similar to renal cancer and BCa, there are no standardized early detection methods for testicular cancer.

## 2. Search Strategy

Our search was guided by predefined inclusion and exclusion criteria, focusing on peer-reviewed articles published between 2006 and 2024, but with a greater emphasis on the publications within the past five years to capture the most current advancements and emerging trends in the field. Search databases included Google Scholar, PubMed, and ScienceDirect.

### 2.1. Inclusion and Exclusion Criteria

The search keywords included all types of genitourinary cancers, oncological therapies (diagnoses, treatments, and surveillances), and dietary, regional, and epidemiological effects. Keywords and Boolean operators were tailored to capture relevant studies, incorporating terms such as “‘genitourinary cancer’, ‘VOCs’, ‘biomarkers’, ‘chromatography’, ‘GC-MS’, and ‘LC-MS’”. Additionally, the search strategy was refined through an iterative process, which included engaging with domain experts and cross-checking references from key publications to uncover additional pertinent articles. Searches were mostly focused on human studies, while animal studies and meta-data studies were also included within the filtering search. We focused on the study population, the number of detected biomarkers (cross-references in other publications), the quality and interpretation of statistical analyses, combined analytical techniques, and the number of biospecimens compared.

### 2.2. Data Extraction and Synthesis

All identified studies were screened in two stages: (1) reviewing titles and abstracts to exclude irrelevant or duplicate entries, and (2) through a full-text review to evaluate alignment with our literature review objectives. Articles were included if they met specific criteria, such as addressing statistical data, point-of-care utilization, biomarker/VOC discovery, access to original research or comprehensive reviews, and English publications. This systematic review aimed to determine the use of VOCs within genitourinary cancer detection and diagnosis. After screening the abstracts and full texts, 200 studies were considered. Of these, 64 studies were excluded for not meeting the criteria. Finally, 136 articles met our inclusion criteria and were included in the systematic review. This rigorous methodology enabled us to systematically identify, evaluate, and synthesize quality evidence to address the core questions of the literature review.

## 3. VOCs as Cancer Markers for Genitourinary Cancers

In search of alternative cancer early detection methods, ongoing research has adopted “omics”, e.g., transcriptomic, proteomic, metabolomic, and lipidomic, approaches to analyze potential cancer biomarkers, which have also led to the discovery of volatile organic compounds (VOCs) [39,40,41,42,43]. Notably, VOCs have been considered to be noninvasive and promising biomarkers, as they can be detected in various biospecimens (i.e., urine, feces, breath, oral, and sweat) [44,45,46,47,48,49]. The overall processes of VOCs internally developed and emitted has been hypothesized to stem from the body’s physiological processes and pathways; thus, VOCs biomarkers have proven to be viable in detecting various diseases [45,50]. Due to the alterations of the disease in normal environments, VOC concentrations in the body could be elevated or suppressed in cancer patients as compared to healthy individuals [47,51,52]. VOC analysis is generally cost-effective compared to traditional diagnostic techniques, such as imaging, invasive biopsies, or even blood testing [53].

**Figure 1:**
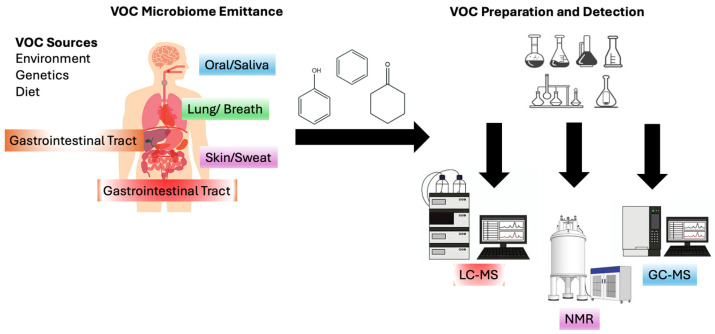
Human-generated sources of volatile organic compounds for potential cancer detection and diagnosis.

The exploration and discovery of VOCs as potential genitourinary cancer biomarkers have gained significant attraction in recent years, with researchers increasingly focusing on the analysis of biospecimens from patients with various cancer types (Figure 1). There is a large variety of literature proposing the use of urine, serum, and tissue samples as potential genitourinary cancer screening biomatrices [40,50,52,54,55,56]. This review will discuss applications of VOCs in three of the major genitourinary cancers—renal, prostate, and bladder—and the clinical impact of target biomarkers as they relate to cancer diagnoses. Additional applications of VOCs in other types of cancer will also be included to highlight the roles of VOCs in cancer biomarker discovery.

### 3.1. VOCs in Tissue for Genitourinary Cancer Detection

The study of tissue VOCs is emerging as a promising avenue for cancer diagnosis and detection due to being the most upstream originator of potential VOCs cancer markers. These VOCs are the byproducts of various metabolic and biochemical processes that occur in both healthy and cancerous tissues, where the unique metabolic and biochemical changes associated with cancer cells can result in the production of distinct VOC profiles [39,57,58]. Tissue VOC profiles can reflect unique metabolic and biochemical changes associated within different cancer types and stages, allowing for the identification of specific biomarkers indicative of disease. Even though it still presents challenges as the most invasive detection technique, this application is balanced by being the most sensitive method for cancer diagnosis [59,60]. Nizioł et al. explored the metabolomic and elemental profiles of kidney urine and tissue to evaluate potential discriminatory VOCs using various analytical techniques. The authors identified five potential tissue VOCs capable of distinguishing cancer and healthy cohorts: sarcosine, fumarate, leucine, xanthine, and tryptophan [61]. Cacciatore et al. investigated the effects of formalin-fixed and paraffin-embedded (FFPE) preservation/storage on prostate cancer tissue metabolic profiles [62]. This study not only presented a unique study of the common storage method, but also reflected the effects of the storage method on the metabolic profile of tissue. The authors stated that FFPE could preserve fatty acids but experienced the greatest loss of peptides, and they suggested that this retrospective storage method of FFPE is most beneficial to metabolomics, with some potential for proteomics [62]. Another study conducted by Zhang et al. looked into the discrimination of renal cell cancer subtypes compared to normal kidney tissues [63]. Within the 71 patient samples, predictive models were able to discriminate the three subtypes with an accuracy of 99.47%. This study is one of many that use analytical techniques and statistical modeling to improve cancer subtype classification and the identification of treatment targets.

Researchers have aimed to identify specific VOC patterns or biomarkers indicative of different types and stages of cancers. While the field of tissue VOC analysis for cancer diagnosis is still in the research and developmental stage, promising results have been reported for various cancer types, including prostate, kidney, and even lung, breast, and colorectal cancer [57,58,64,65,66,67]. However, further research and the standardization of techniques are needed to establish the clinical utility of tissue VOC analysis for widespread cancer diagnosis. As this field of research continues to evolve, tissue VOC analysis holds promise as a highly sensitive tool for cancer diagnosis and management.

### 3.2. VOCs in Blood for Genitourinary Cancer Detection

Blood samples are relatively easy to collect, eliminating the need for painful, expensive, and risky biopsy procedures. Thus, it can be performed routinely to identify cancer-specific VOC profiles in blood samples for early diagnosis, monitoring of treatment responses, and even predicting disease recurrence [68,69,70]. Additionally, while the non-specific symptoms of early-stage cancer can make diagnosis challenging, blood VOC analysis may help improve accuracy and reduce the risk of false-negative results [71,72].

Maslov et al. utilized a metabolomic approach to study the early detection of renal cancer in plasma. The results indicate an accuracy of 70% using a model constructed with ten metabolites (citrate, glutamate, arginine, tyrosine, phenylalanine, methionine, tryptophan, pipecolinic acid, lysoPC (20:5), and PC (32:2) (phosphatidylcholines) [73]. This research can potentially expand and identify renal-specific profile changes to be used in early diagnosis with high accuracy.

VOCs in blood for genitourinary cancer have been quite limited. However, beyond genitourinary cancer detection, Ly-Verdu et al. investigated blood profiling on the changes in liver metabolites using both metabolomic and proteomic approaches. This inter-comparison provides insight into the application of the different “omic” approaches that can lead to the development of greater sensitivity, reliability, and understanding of VOC applications. The research team used perfused and unperfused mice liver tissue to explore the potential overlap between blood and tissue profiles [74]. As a result, the authors observed 17 metabolites with significant peak area changes between both biospecimens. Also, quantitative differences within metabolite concentrations are an important field to study, as it relates to VOC biomarker discovery.

The applications of blood VOC analysis extend beyond cancer diagnosis. This approach can also be used for risk assessment, as certain individuals may have genetic or lifestyle factors that predispose them to specific cancer types [75]. Monitoring changes in blood VOC profiles over time can provide valuable information about an individual’s risk and the effectiveness of cancer prevention strategies [51]. Furthermore, blood VOC analysis has the potential to play a role in personalized medicine by guiding treatment decisions and helping healthcare providers tailor therapies to the specific characteristics of the patient and their cancer. While research and validation are ongoing, blood VOCs show great potential in enhancing cancer diagnosis and management, ultimately contributing to improved patient outcomes.

### 3.3. VOCs in Feces for Cancer Detection

The unique metabolic and biochemical processes associated with cancer can result in altered VOC profiles in the stomach, and these changes can be indicative of various gastrointestinal and even intra-intestinal cancers [57,76,77,78]. By examining fecal VOCs, researchers aim to identify specific biomarkers or patterns that can serve as reliable indicators of the presence of cancer. Fecal biospecimens have not yet been extensively researched concerning genitourinary cancers, representing an area with potential for further exploration. While studies investigating VOCs in fecal samples have primarily focused on colorectal cancer, they reveal several VOCs that could also be relevant for genitourinary cancers due to metabolic overlap. Many of these compounds are involved in inflammation, oxidative stress, and metabolic pathways common to various cancers, suggesting that fecal VOC analysis might offer insights into genitourinary malignancies as well. Expanding research in this area could potentially reveal applicable biomarkers across different cancer types, enhancing early detection and diagnostic strategies.

As mentioned above, fecal VOCs are gaining attention for their potential benefits and applications in cancer diagnosis. Śmiełowska et al. research consisted of a 35-patient cohort looking at the comparison of colorectal cancer and healthy patients where they investigated the gaseous form released from breath and fecal samples. The researchers observed elevated amounts of heptanoic acid, acetone, 2,6,10-trimethldodecane, n-hexane, skatole, and dimethyl trisulfide within cancer patients. The accuracy of the diagnostic model performed above 90%, indicating that breath and fecal samples are viable in the search for VOCs for colorectal cancer [53].

Additionally, fecal VOC analysis has the potential to detect not only gastrointestinal cancers but also other systemic cancers, as systemic effects of cancer can influence stomach VOC profiles. This widens the scope of potential applications, allowing for comprehensive and multi-cancer screening [78,79]. Ongoing research is investigating its utility for other types of cancer, including stomach, pancreatic, and even lung cancer, due to the systemic effects of malignancies on the stomach microbiome. Unlike traditional diagnostic methods that may require invasive procedures, such as tissue biopsies or endoscopy, collecting a stool sample is a simple and patient-friendly process. This makes it more acceptable for cancer screening and may encourage a broader population to undergo regular testing, ultimately facilitating earlier cancer detection and intervention. As the detection techniques of this biospecimen increase, standardized testing protocols will need to be developed so that fecal VOC analysis will have the potential to become a valuable tool in the early detection and monitoring of various cancer types.

### 3.4. VOCs in Urine for Genitourinary Cancer Detection

Urine is the most collected downstream biospecimen. Over the years, urine has gained significant attention from clinical researchers as a noninvasive and highly informative tool for cancer diagnosis and monitoring. One of the key benefits of using urine VOCs is the ease of sample collection and the voided volumes. Unlike more invasive methods such as tissue biopsies or even blood testing, urine can be collected with minimal discomfort to the patient, making it a practical and widely accepted diagnostic medium. This noninvasive nature of urine VOC analysis can encourage more frequent and routine cancer screenings, potentially leading to earlier cancer detection and decrements in late-stage diagnoses.

Costantini et al. investigated urinary volatilomes of 142 healthy controls and 110 renal cancer patients using a commercialized electric nose (Cyranose 320). The research team was able to correctly identify patients with an accuracy of 85%, a sensitivity of 71.8%, and a specificity of 89.4%. Urinary VOC profiling using e-Noses has been proven accurate, portable, and noninvasive, which has the potential of being adopted in clinical diagnostic applications. However, it is notable to mention that one of the drawbacks of e-Noses is the reduction in specificity and sensitivity compared to traditional mass spectrometry techniques. Also, this analytical technique presents the inability to identify and quantify specific VOCs, thus only providing the overall scent signature or signals of the VOCs [80]. Ultimately, more vigorous research is needed to support using solely electronic noses in the clinical setting for accurate cancer diagnosis and monitoring. Bax et al. performed a comprehensive review from 2003 to 2019, focusing on the diagnosis, prognostic, and predictive value of the specific metabolites within various cancers and matrices, suggesting that the VOCs prevalent within RCC included acetylcarnitine and additional carnitine derivatives attributed to carnitine and fatty acid oxidation pathways [17]. Pastore et al. reviewed urine and serum, proposing potential risk factors, classifications, and treatments of RCC.

Urinary biomarkers also show promising diagnostic power for PCa. Gao et al. [56] developed a urinary VOC-based model for PCa detection. Using linear regression, the area under the receiver operating characteristic curve (AUC) for the model was 0.92 (sensitivity, 0.96; specificity, 0.80) in the training set and 0.86 in the testing set. Kalid et al. [81] developed a model based on PSA and 4 VOCs, 2,6-dimethyl-7-octen-2-ol, pentanal, 3-octanone, and 2-octanone. A mean accuracy of 74% was accomplished when using Random Forest.

Ligor et al. found potential biomarkers in urine for BCa [82]. Urinary VOCs such as butyrolactone, 2-methoxyphenol,3-methoxy-5-methylphenol, 1-(2,6,6-trimethylcyclohexa-1,3-dien-1-yl)-2-buten-1-one, nootkatone, and 1-(2,6,6-trimethyl-1-cyclohexenyl)-2-buten-1-one were found significantly elevated in PCa patients and considered promising candidates for VOC model BCa biomarkers in urine. Lett et al. [83] studied VOCs profiles in 305 BCa positive and negative subjects and found that ten urinary VOCs (nonanal, 2-ethylhexan-1-ol, 1,1,4a-trimethyl-4,5,6,7-tetrahydro-3H-naphthalen-2-one, 5-ethyl-3-methyloxolan-2-one, phenol, 4-methylpent-3-enoic acid, 2-methoxyphenol, 3-methylheptan-2-one, 1,2,4,5-tetramethylbenzene, and Heptan-2-one) were significantly different between the cancer-positive and -negative groups. The group further developed an eight-VOC diagnostic biomarker panel (combined nonanal, phenol, 5-ethyl-3-methyloxolan-2-one, 2-ethylhexan-1-ol, 1,1,4a-trimethyl-4,5,6,7-tetrahydro-3H-naphthalen-2-one, 1-methyl-4-propan-2-ylcyclohexan-1-ol, benzaldehyde, and 2,6-dimethyloct-7-en-2-ol) and achieved AUC 0.77 (sensitivity 0.71, specificity 0.72).

Pinto et al. explored the identification of VOCs in urine as potential biomarkers for non-invasive bladder cancer diagnosis and staging using HS-SPME-GM-MS [84]. The researchers analyzed urine samples from patients with bladder cancer and healthy controls. The study identified a distinct panel of VOCs, including specific aldehydes, ketones, and hydrocarbons, that were significantly elevated in the urine of bladder cancer patients compared to controls. Among the key findings, VOCs such as 2-butanone and 4-heptanone showed strong discriminative power, with sensitivities and specificities exceeding 85% for distinguishing cancer stages. These biomarkers also demonstrated potential for stratifying patients into early- and late-stage disease, offering insights into tumor progression. The study underscores the promise of urine metabolomics as a non-invasive diagnostic tool for bladder cancer, highlighting its potential for improving early detection and personalized patient management.

Another study conducted by Pinto and colleagues utilized urinary volatilome profiling of 75 ccRCC patients and 75 cancer-free controls to identify a volatile signature characteristic of ccRCC [85]. Using HS-SPME-GC-MS, the analysis revealed 22 significantly altered volatile metabolites, including aldehydes, ketones, aromatic hydrocarbons, and terpenoids. A six-biomarker panel (octanal, 3-methylbutanal, benzaldehyde, 2-furaldehyde, 4-heptanone, and p-cresol) demonstrated strong discriminatory power with 83% sensitivity, 79% specificity, and 81% accuracy. The ccRCC volatilome signature highlighted dysregulated energy metabolism and carcinogenesis-related enzymatic overexpression, providing a foundation for the development of bioelectronic sensors for ccRCC detection.

A study by Lima et al. focused on the potential of urinary VOCs as biomarkers for the differential diagnosis of prostate cancer (PCa) from other urological cancers (bladder (BC) and renal (RC)) [86]. Using GC-MS, the authors analyzed urine samples from 80 male patients, comprising of 20 PCa, 20 BC, 20 RC, and 20 healthy controls. Statistical analysis, including principal component analysis (PCA) and partial least squares-discriminant analysis (PLS-DA), revealed distinct VOC profiles for each cancer type. A panel of six VOCs demonstrated high diagnostic accuracy for differentiating PCa from BC and RC with an area under the curve (AUC) of 0.92. The sensitivity and specificity for PCa diagnosis were 76% and 90%, respectively. This study highlights the potential of urinary VOC profiling as a non-invasive diagnostic tool to enhance the specificity and accuracy of PCa diagnosis compared to other urological cancers.

Beyond genitourinary cancer detection, Wen et al. explored urinary volatolomics to evaluate healthy and pancreatic ductal adenocarcinoma (PDAC) patients; this untargeted study identified four potential discriminatory biomarkers: 2-pentanone, hexanal, 2-hexanone, and *p*-cymene. The researchers were able to accurately discriminate between both cohorts, supporting the application of urinary VOCs in cancer biomarker discovery [87].

### 3.5. VOCs in Breath for Cancer Detection

Lastly, breath VOCs in cancer diagnosis are in their adolescent stage but are gaining more recognition and hold great promise in the field of cancer diagnosis. One of breath’s key advantages is its noninvasiveness, making it a patient-friendly method for cancer detection. The applications of breath VOCs in cancer diagnosis are diverse and continue to expand. On-going research focuses on developing sensitive and specific techniques for detecting cancer-related VOC patterns. These patterns can distinguish between different cancer types and stages, aiding in early diagnosis and personalized treatment plans [44]. Nonetheless, the application of breath VOCs still faces the challenge for standardized sampling and analysis methods, as different studies often yield inconsistent results due to variations in breath collection techniques, patient conditions, and analytical instruments.

The research on using breath volatile organic compounds (VOCs) for diagnosing genitourinary cancers—such as prostate, bladder, and kidney cancers—remains limited and less developed compared to studies on other cancer types like lung and gastrointestinal cancers. Markar et al. focused on exhaled breath for esophagogastric cancer diagnosis within 335 healthy and cancer patients. The results of the experiment present a five VOC diagnostic model (butyric acid, pentanoic acid, hexanoic acid, butanal, and decanal) capable of discriminating both cohorts with a diagnostic accuracy of 85% [88]. Nakhleh et al. identified 17 diseases via breath pattern analysis; this cohort consisted of 1404 patients from five different countries and diseases ranging from lung cancer to Parkinson’s. The cohort represented a comprehensive approach to the human population. The research team utilized artificially intelligent nanoarray coupled with gas chromatography to identify each breath pattern, which identified 13 VOCs that could distinguish between each disease. Overall, breath VOC analysis offers an inexpensive and noninvasive technique for disease detection and diagnosis [89].

Breath VOC analysis can also help monitor treatment effectiveness and enable timely adjustments to therapy regimens. Moreover, it has the potential to serve as a screening tool for high-risk populations, allowing for earlier interventions in those with an increased likelihood of developing cancer, such as smokers or individuals with a family history of the disease. As this field continues to evolve, developing portable and user-friendly devices for breath analysis may facilitate its integration into routine clinical practice, ultimately improving the efficiency and effectiveness of cancer diagnosis and management. Within breath analysis, there is still a dire need for standardization and validation of the technical challenges geared towards continual enhancement to be adopted by clinical applications [90]. A list of VOC cancer biomarkers is shown in Table 1.

### 3.6. Other “Omics” in Genitourinary Cancer Diagnosis

The recent literature has increased the number of manuscripts published within the “omics cascade” approaches including genomics, transcriptomics, proteomics, and metabolomics. Several reports have analyzed microRNAs in urine and blood for prostate cancer, which has allowed for diagnostic performance to be warranted [91,92]. Transcriptomics presents drawbacks in the lack of gene identification within complex and adaptive response environments, warranting advancement in detection and diagnostic measures [93].

Proteomics addresses large-scale proteomes, proteins, and peptides; this approach can provide information on protein expression, function, structure, protein–protein interactions, and post-transcriptional modifications. Common analytical techniques for proteomics coupled with mass spectrometry include liquid chromatography (LC-MS), Western blot, capillary electrophoresis (CE-MS), surface laser desorption/ionization (SELDI-TOF-MS), and enzyme-linked immunoassay (ELISA). Sun et al. utilized comparative proteomic profiling to detect and suggest prognostic factors specific to clear cell renal cell carcinoma (ccRCC) within renal tissue [94]. Johaan et al. presented a novel method for identifying cancer biomarkers through combined blood and tissue analysis in RCC. Using a subtractive tissue-directed shotgun proteomics approach, the group detected specific tumor proteins in the blood that are also present in cancerous tissue [95]. By comparing the tumor, adjacent normal tissue, and plasma samples from a single patient, the team identified unique proteins that were more prevalent in tumor tissue than in normal tissue, which were also detectable in the blood [71,73,74,95]. These proteins—such as cadherin-5, cadherin-11, and pyruvate kinase—were further validated using Western blot analysis in additional RCC patients, supporting the utility of this approach for discovering potential biomarkers. This study highlights the promise of integrating blood and tissue analysis to enhance the early detection and diagnosis of RCC and possibly other cancers.

Urinary protein biomarkers were reported when comparing patients with RCC and a control group which included kidney injury molecule-1 (KIM-1), aquaporin-1 (AQP-1), and perilipin 2 (PLIN2). Kim-1 has been proven sensitive for RCC; however, it lacks reliable specificity within RCC detection due to its relevance found in various acute and chronic kidney diseases and injuries [54]. An overexpression attributed to AQP-1 and PLIN2 within renal tumor tissue surgical resection indicates that the excretion of these upregulated proteins has been found to be elevated within ccRCC and pRCC patients. Both biomarkers presented high sensitivity (AQP-1 = 100%; PLIN2 = 92%) and specificity (AQP-1 = 100%; PLIN2 = 100%) [13]. These proteins have showcased no significant influx when differentiating between noncancerous kidney diseases; warranting focused research and reliability for AQP-1 and PLIN2 detection in RCC [54].

Radiogenomics is increasingly being explored for genitourinary cancers, particularly in prostate, kidney, and bladder cancers. Radiogenomics integrates imaging data with genetic or molecular information to help identify cancer-specific characteristics, allowing for more personalized treatment approaches. For instance, in prostate cancer, MRI-based radiogenomic approaches can correlate specific imaging features with genetic markers like BRCA mutations or androgen receptor pathways, which can indicate more aggressive cancer types and guide treatment decisions. In renal cell carcinoma, radiogenomics is used to associate CT (Computed Tomography) and MRI features with specific genetic mutations, such as VHL (von Hippel–Lindau) mutations, which are common in RCC [96,97,98]. These correlations can help differentiate between subtypes of RCC, potentially aiding in treatment planning and predicting patient outcomes. For bladder cancer, although radio genomics is less developed, studies are beginning to assess how imaging characteristics relate to mutations in genes like FGFR3, which are relevant to tumor progression and may inform tailored therapeutic options. Radiogenomics holds promise for advancing precision medicine in genitourinary cancers, although more research is needed to standardize techniques and validate biomarkers across larger, diverse patient cohorts. As data availability grows and machine learning algorithms improve, radiogenomics may become a valuable tool in routine clinical practice for these cancers [98,99]. While radiogenomics holds promise for personalized non-invasive cancer care, overcoming technical, financial, and ethical barriers will be essential to broader adoption in clinical settings.

Significant development has been made in genetic and genomic testing for genitourinary cancer as well. Germline genetic variants (e.g., BRCA2 or MSH2/6) and polygenic germline risk scores are being investigated in PCa screening and prognosis [100]. Mutations in FGFR3 or HRAS are found in 65–80% of low-grade BCa cases and are less frequent in high-grade tumors [101]. Circulating tumor DNA (ctDNA) in blood or urine is also recognized as a non-invasive method of identifying and monitoring genitourinary cancers, including prostate [102], bladder [103], and renal cell carcinoma [104]. Understanding these genomic alterations could be valuable in recognizing the biology in genitourinary cancer.

## 4. Extraction and Detection of VOCs

**Figure 2:**
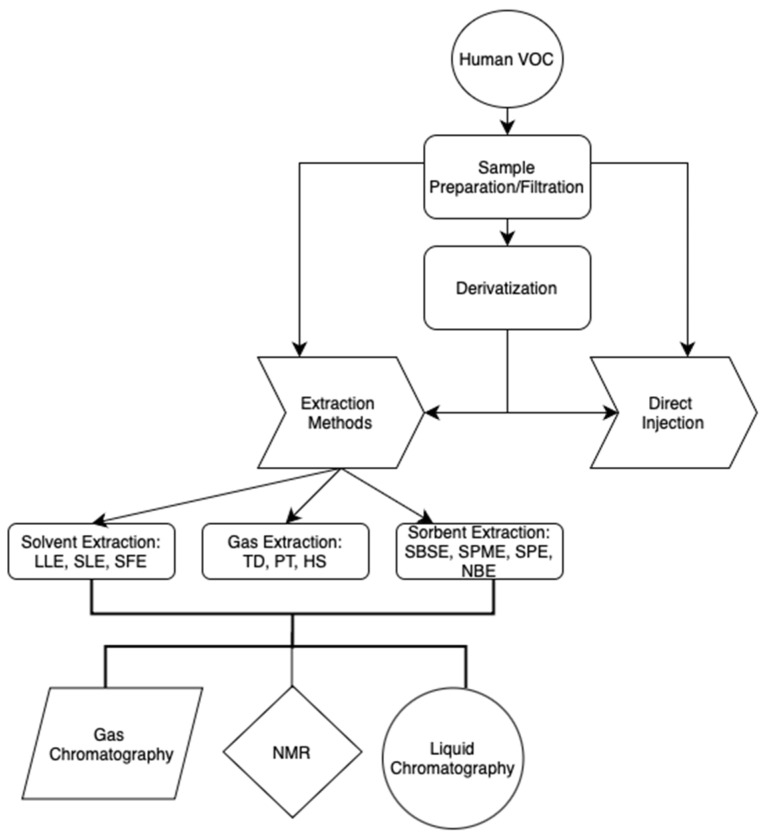
Human VOC sample extraction methods flowchart.

Most VOCs within biological matrices are found at low concentrations ranging from parts per trillion to parts per million, and that adds emphasis to the preconcentration step prior to analysis. This preconcentration process is often the most labor-intensive part of the analysis and is the primary source of errors that can impact the accuracy and reliability of the results. To enhance reproducibility and reduce interference from other compounds, minimizing the steps in the sample preparation process is crucial. An ideal sample preparation technique should possess the qualities of, but not be limited to, simplicity, high extraction capacity, selectivity, efficiency, speed, potential for automation and miniaturization, compatibility with a range of separation and detection methods, and safety for both the operator and the environment. We discuss some of the primary extraction and detection methods used to detect VOCs in breath, blood, and other bodily fluids for in-cancer detection, with an emphasis on genitourinary cancers. Figure 2 summarizes the VOC extraction workflow. 

### 4.1. Extraction Methods

Solid-phase microextraction (SPME) is a widely utilized technique for VOC extraction. It involves the adsorption of VOCs onto a solid-phase fiber, followed by desorption and analysis. Mochalski et al. highlighted the effectiveness of SPME in collecting VOCs from breath samples and its potential applications in cancer diagnosis [105,106,107]. Solid-phase microextraction coupled with gas chromatography has found important applications in the extraction of VOCs for the detection and diagnosis of genitourinary cancers, including bladder, kidney, prostate, and testicular cancers [7,9]. SPME is particularly well-suited for urine as a sample matrix. SPME can be used to capture and concentrate VOCs from urine samples directly, allowing for the identification of potential cancer biomarkers. In genitourinary cancer diagnosis, SPME is applied to analyze urine samples collected from patients by exposing a coated SPME fiber to the urine sample; VOCs present in the urine are adsorbed onto the fiber. After a specified extraction time, the SPME device is removed from the sample and thermally desorbed to release the captured VOCs [105]. The ability of SPME to collect and concentrate VOCs from urine and breath [108] makes it a valuable tool for the potential early detection and monitoring of genitourinary cancers, offering the possibility of a less invasive and more frequent screening method. However, as with any diagnostic technique, extensive research and validation are necessary to confirm the reliability and clinical utility of SPME in genitourinary cancer diagnosis.

Like SPME, stir bar sorptive extraction (SBSE) is a sample preparation technique used in cancer diagnosis to extract and concentrate VOCs from various biological samples, such as blood or urine. For example, in the case of prostate cancer, SBSE can be utilized to capture VOCs from urine samples, providing a means for noninvasive detection and monitoring [45,56,109]. SBSE coupled with GC-MS was also used to study urinary VOCs for RCC diagnosis [110]. Additionally, SBSE can be applied in the study of bladder cancer, where the analysis of urine VOCs using SBSE may offer a noninvasive way to identify specific patterns associated with the disease. Overall, SBSE holds the potential in metabolomic study and provides a promising avenue for a robust and noninvasive cancer detection and biomarker discovery platform [111,112,113].

Berrou et al. compared SBSE to SPME in obtaining volatile and semi-volatile metabolic profiles of *Staphylococcus aureus*. The group was able to identify 12 VOCs, with a greater concentration and sensitivity of SBSE compared to SPME. In the primary comparison of SPME and SBSE, SBSE typically provides higher extraction efficiency due to the larger sorbent phase (the external stir bar coating) presenting the ability to capture larger quantity of VOCs, which can be particularly advantageous when dealing with low concentrations or complex sample matrices [114]. SBSE can handle larger sample volumes of complex biological samples like urine or blood, offering a greater potential for VOC extraction. When dealing with SBSE’s sample preparation involves continuous stirring or agitation of the sample during extraction, ensuring an even distribution of VOCs onto the sorbent coating [109]. A benefit of continuous stirring is that it minimizes variations in extraction time and sample–sorbent interactions, leading to enhanced reproducibility in VOC detection. This is very important for reliable and consistent results in clinical diagnosis. Furthermore, SBSE offers adaptability to high-matrix samples, where the matrix complexity can interfere with VOC extraction; the larger sorbent phase in SBSE can better handle and accommodate these complex matrices [109,115]. The choice between SBSE and SPME depends on the specific analytical requirements and characteristics of the sample matrix. Researchers and analysts should consider factors such as the type of sample, the target analytes, sensitivity, and the available equipment when selecting the most suitable extraction method for their applications. It should also be noted that both SBSE and SPME are considered green sample preparation techniques, as no or minimal use of harmful solvents are used in the process [116,117].

Lastly, needle-based extraction (NBE) is particularly useful for collecting VOCs directly from cancerous tissues or tumors. Needle-based extraction is innovative and offers a minimally invasive technique compared to traditional biopsy procedures and plays a crucial role in the sample collection and detection of cancer. This method involves the insertion of a specialized needle, equipped with a sorbent or coating at the tip into the sample of interest for the direct extraction of VOCs. Needle-based extraction offers several key advantages, making it a valuable tool in different fields. One of the primary benefits of needle-based extraction is its ability to obtain VOCs directly from specific sites of interest, such as tumors or tissues. This approach allows for real-time analysis and provides a unique opportunity to investigate VOC profiles directly at the source of potential diseases like cancer. NBE minimizes the risks of contamination and interference from surrounding matrices, thus enhancing the accuracy and reliability of the VOC analysis. Moreover, NBE can be utilized in surgeries or medical procedures, making it a valuable tool for intraoperative diagnosis and the assessment of tumor margins. In cancer research, needle-based extraction has shown promise for analyzing tissue VOCs, aiming to identify specific patterns or biomarkers associated with different cancer types and stages [118,119,120,121]. By capturing VOCs directly from tumor sites, researchers can gain insights into the metabolic and biochemical changes within cancer tissues, potentially leading to the discovery of novel diagnostic and prognostic markers. This method also holds the potential for real-time treatment response monitoring and residual disease assessment during surgical procedures, offering a dynamic approach to improving cancer management; however, NBE is highly invasive. Porto-Figueira et al. explored needle trap microextraction to discriminate urinary VOCs among breast and colon cancer. The research team was able to identify 130 VOCs, which were further discriminated against using statistical analysis [118]. Needle-based extraction’s adaptability to various sample types and its potential for early disease detection makes it a promising and versatile technique in the field of healthcare and biomedical research [120].

### 4.2. Detection Methods

Gas chromatography–mass spectrometry (GC-MS) is a well-established technique for the separation and identification of VOCs. GC-MS combines two distinct components: gas chromatography for separating complex mixtures of VOCs, and mass spectrometry for identifying and quantifying individual compounds based on their mass-to-charge ratios. This analytical approach allows for researchers to analyze the intricate VOC profiles found in biological samples like breath, blood, or tissues, seeking to identify specific compounds associated with cancer [50,56,63,79,87]. Researchers utilized a GC-MS metabolomics-based approach in kidney [110,122], prostate [56,81], and bladder [82,83] cancers, as well as in patients’ urine to identify a potential diagnostic biomarker panel. GC-MS can identify these VOCs, offering insights into the early detection, diagnosis, and monitoring of cancer. Additionally, GC-MS provides high sensitivity and specificity, presenting the capability of detecting even trace amounts of VOCs that may be indicative of cancer. Furthermore, GC-MS can be used in combination with advanced statistical and machine-learning techniques to process large datasets and facilitate the identification of specific VOC patterns associated with different cancer types and stages. As a result, GC-MS plays a pivotal role in advancing cancer biomarker discovery and has the potential to significantly impact early diagnosis and treatment outcomes in the field of oncology [123,124].

Liquid chromatography–mass spectrometry (LC-MS) is another common determinative technique for cancer biomarker discovery. In cancer research, LC-MS is applied to analyze a wide range of biological samples, such as blood and urine, to identify VOC patterns indicative of the targeted disease [60,63,72,108]. LC-MS allows for the comprehensive analysis not only of known VOC biomarkers, but also of novel compounds that may be associated with specific cancer types or stages [73]. The use of LC-MS in cancer research continues to expand our understanding of the disease and holds great promise for improving cancer diagnosis and management [125,126,127].

Sreekumar et al. used a combination of high-throughput liquid–chromatography-based mass spectrometry to detect over 1000 metabolites in tissues, urine, and plasma samples. The metabolomic profiles were able to distinguish benign prostate, clinically localized prostate cancer, and metastatic disease [128]. In their study, sarcosine was identified as a biomarker that was highly increased during prostate cancer progression to metastasis and can be detected non-invasively in urine. Lin et al. applied a combination of LC−MS analyses of metabolites in blood samples with multivariate statistical analyses for RCC diagnosis, and this has potential in the staging of RCC [129]. Luczykowski et al. evaluated the urinary metabolomes of bladder cancer patients using SPME-LC-MS. Chemometric or VOC analysis selected various metabolites which aided in the distinction between healthy and bladder cancer patients [107]. There was an observed significant difference between phenylalanine-metabolized compounds and metabolic pathways which involve histidine, beta-alanine, and glycerophospholipids.

Sample derivatization is often necessary for cancer detection when the target analytes are chemically complex, unstable, or present in low concentrations, making them difficult to detect using standard analytical techniques. By introducing a derivatizing agent, specific functional groups of the analytes are modified to enhance their stability, volatility, and detectability, allowing for more accurate and sensitive measurements. This process is particularly crucial for VOCs and metabolites in biological samples, where derivatization can improve chromatographic separation and mass spectrometric analysis. In cancer detection, derivatization facilitates the identification and quantification of biomarkers that may be otherwise challenging to detect, thus enhancing the reliability and sensitivity of diagnostic methods such as GC-MS or LC-MS.

These extraction and detection methods, often combined with machine learning and pattern recognition techniques, represent a dynamic and evolving frontier in the field of cancer diagnosis. The use of comprehensive analytical techniques, SPME, SBSE, and NBE coupled with GC-MS and LC-MS, allows for the capture and analysis of VOCs from various biomatrices. Moreover, the application of machine learning algorithms enhances the diagnostic power of VOC-based cancer detection by enabling the identification of complex VOC patterns associated with different cancer types and stages [123,124,130,131,132,133]. A comparative list of VOC extraction and detection methods is shown in Table 2, whereas Table 3 provides the literature support for various uses of analytical methods to detect VOCs.

## 5. Perspectives and Future Recommendations

The exploration of volatile organic compounds (VOCs) as potential biomarkers for genitourinary cancers is a rapidly evolving field, yet several challenges and opportunities remain to realize their clinical potential fully. The specificity of VOC profiles to cancerous states is driven by the metabolic dysregulation inherent to malignancies, presenting a valuable avenue for early detection and personalized treatment strategies. However, the field is still in its early stages, with challenges related to standardization, reproducibility, and the influence of confounding factors such as diet and comorbidities. Addressing these challenges requires refining detection methods and validating VOC signatures in large, diverse patient cohorts.

While the previous literature has demonstrated the diagnostic promise of VOCs, future research should focus on improving the specificity and sensitivity of detection methods. Future research should also focus on integrating advanced analytical techniques, such as high-resolution mass spectrometry and gas chromatography, as well as electronic nose systems with robust machine-learning algorithms, to enhance the detection and interpretation of VOCs. These additions are necessary to achieve high-throughput, field-deployability (point of care), non-invasive screening tools that can accurately differentiate cancer-specific metabolomic signatures. Furthermore, standardization in sample collection, storage, and analysis protocols is critical to ensure reproducibility and comparability across studies.

Detection methods should be a critical focus; gaining deeper insights into the underlying mechanisms of VOC production in genitourinary cancers could significantly enhance biomarker specificity. Research into how tumor metabolisms influence VOC profiles may uncover new biomarkers and improve the differentiation between malignant and benign conditions. Integrating multi-omics approaches, including metabolomics, genomics, and proteomics, could provide a comprehensive understanding of the biological underpinnings of VOCs and their diagnostic potential. Interdisciplinary collaboration will play a pivotal role in advancing this field. Partnerships between oncologists, analytical chemists, bioinformaticians, and engineers can drive the innovation of detection platforms tailored for clinical use. Integrating machine learning and artificial intelligence into VOC data analysis could uncover subtle patterns and correlations, improving diagnostic accuracy. Moreover, combining VOC analysis with other biomarker modalities, such as genomics and proteomics, may yield robust multimodal diagnostic approaches that increase reliability and reduce false positives. A key area for future work involves the development of large-scale multicenter studies to validate the diagnostic and prognostic utility of VOCs in diverse patient populations. Current studies often suffer from small sample sizes and limited demographic diversity, which can restrict the generalizability of findings. Expanding research to include varied genetic, environmental, and lifestyle factors will enhance our understanding of how these variables influence VOC profiles. Additionally, longitudinal studies are required to evaluate the utility of VOCs in monitoring disease progression, treatment response, and recurrence, providing a comprehensive picture of their clinical applications.

Finally, regulatory and ethical approvals, cost-effectiveness analyses, and user-friendly interfaces must be addressed to facilitate the transition of VOC-based technologies from research to clinical practice. Developing clear guidelines for biomarker validation, data interpretation, and patient privacy will build confidence among stakeholders, including healthcare providers and patients. Investment in public awareness and education about the benefits and limitations of VOC-based diagnostics can also help foster acceptance. By addressing these challenges and leveraging emerging technologies, VOCs as biomarkers for genitourinary cancers hold significant potential for transforming cancer detection and management.

## 6. Conclusions

The field of VOC detection methods has made significant strides in recent years, offering new possibilities for noninvasive genitourinary cancer diagnosis and monitoring. Techniques like SPME, SBSE, NBE coupled with GC-MS, and LC-MS have expanded the toolkit for capturing VOC biomarkers associated with bladder, kidney, and prostate cancer. The innovation of machine learning’s ability to analyze vast datasets, detect patterns, and make predictions shows the potential to significantly improve the accuracy and efficiency of cancer diagnosis, ultimately leading to better patient outcomes, reduced healthcare costs, and advancements in cancer research and treatment.

Research on specific volatile organic compounds (VOCs) associated with testicular cancer is still in the early stages, but several compounds have shown potential as biomarkers. In studies focused on cancer-related VOCs, researchers have identified certain aldehydes, alkanes, and aromatic compounds that may indicate testicular cancer due to their links to altered metabolic pathways in cancer cells [135,136]. For instance, hexanal and heptanal, both aldehydes, are often elevated in cancer patients due to lipid peroxidation, a process more prevalent in cancerous tissues. Other compounds like ethanol, ethylbenzene, and 1-octene have also been noted in preliminary studies as potentially linked to the metabolic changes associated with testicular cancer. Furthermore, VOCs such as 2-propanol and acetone might appear in higher concentrations in patients with testicular cancer, as they are connected to oxidative stress and lipid metabolism alterations often seen in cancer [34,37]. However, it is important to note that these findings are still preliminary, and further research is needed to validate these VOCs as reliable markers for testicular cancer specifically.

Undoubtedly, other “omics”, such as proteomics, are reliable and accurate analytical techniques for cancer diagnosis. However, they require expertise in the construction of databases and experienced technical skills, and often are laborious and costly. These drawbacks suggest that there is still room for their potential use in other analytical techniques for cancer biomarker detection.

In the future, several important directions in genitourinary VOC detection methods will become apparent. Firstly, further research is needed to establish the clinical utility of these techniques, including large-scale clinical trials to validate the identified VOC biomarkers. Secondly, standardization and validation are crucial to ensure the reliability and reproducibility of results in real-world clinical applications. Furthermore, the integration of VOC-based detection methods into routine clinical practice requires the development of user-friendly, minimally invasive, and cost-effective technologies that can be readily adopted by healthcare professionals. Advances in instrumentation, such as portable breath analyzers and real-time monitoring systems, are likely to play a pivotal role in achieving this goal. The ongoing exploration of genitourinary VOCs can contribute to a deeper understanding of cancer biology and the development of targeted therapies. These advancements can lead to personalized treatment strategies based on the unique VOC profiles of individual patients by fostering a new era of precision medicine in the field of genitourinary cancer. Overall, the future holds promise for these innovative VOC detection methods, indicating the transformation of the way genitourinary cancers are detected, characterized, and managed, all with patients’ best interests in mind.

## Figures and Tables

**Table 1:** A summary of VOC biomarkers for genitourinary and other cancers.

Cancer/Biospecimen	Analytical Methods	Detected Biomarkers	Reference
Renal cell carcinoma	Literature review	N/A	Pastore et al. [54]
Human and isogenic prostate cancer cell lines (Frozen/FFPE tissue)	MS and Promega	352–460 markers including fatty acids, peptides, and steroids	Cacciatore et al. [62]
Prostate cancer (Urine)	Literature review	N/A	Bax et al. [17]
Renal cell cancer (Tissue)	DESI-MS	MG (18:1); Cer(d40:1); CL(74:8); PS(38:4); PI(34:1); PI(36:1)	Zhang et al. [63]
Kidney cancer (tumor/tissue)	ICP-OES, LDI MS and 1H NMR	Fumarate; Leucine; Sarcosine; Tryptophan; Phenylalanine; Glucose; Creatine; Zn; S; Na; Hydoxyeicosatrienoic acid; Octanediol; Diethoxypentane; Oxoalanine; 1-(Methyl-thio)ethyl-2-propenyl disulfide	Nizioł et al. [61]
Renal cell cancer (Blood/Plasma)	MS	Pipecolinic acid; Glutamate; Methionine; Arginine; Tyrosine; Phenyalanine; Tryptophan; Citrate	Maslov et al. [73]
Renal cancer (Urine)	Electronic nose	N/A	Costantini et al. [80]
Clear cell Renal cell carcinoma (Urine)	HS-SPME-GC-MS	22 significantly VOCs including aldehydes, ketones, aromatic hy-drocarbons, and terpenoids. A six-biomarker panel (octanal, 3-methylbutanal, benzal-dehyde, 2-furaldehyde, 4-heptanone, and p-cresol) demonstrated strong discriminatory power	Pinto el al. [85]
Mice Liver (Blood/Tissue)	GC x GC-TOFMS and LC-MS/MS	Malic acid; *cis*-4-Hydroxy-L-proline; Fructose-6-phosphate; Citric acid; Aspartic acid; Tyrosine; Mannonic acid lactone; Threonic acid-1,4-lactone; Linoleic acid; Inositol; Valine; Pyroglutamic acid; Squalene; Urea	Ly-Verdu et al. [74]
Prostate cancer (Urine)	TD-GC-MS	4-(3,4-dihydro-2,2,4-trimethyl-2H-1-benzopyran-4-yl)-phenol, Estradiol, Ethyl à-hydroxymyristate trisiloxane, 1-(2,4-Dimethylphenyl)-3-(tetrahydrofuryl-2)propane, 2-amino-Imidazole-5-carboxylic acid, 1,1,3,3,5,5,7,7,9,9-decamethyl-pentasiloxane, 1,1,1,5,5,5-hexamethyl-3,3-bis[(trimethylsilyl)oxy]-, Trisiloxane, Phthalic acid, bis(7-methyloctyl) ester, 4-Nitro-4′-chlorodiphenylsulfoxide, 1-Propylpentachlorotriphosphazene, 2,6-di-t-butyl-4-hydroxymethylene-2,3,5,6-detetrahydrocyclohexanone	Gao et al. [56]
Prostate cancer (Urine)	SPME-GC-MS	2,6-dimethyl-7-octen-2-ol, pentanal, 3-octanone, and 2-octanone	Khalid et al. [81]
Prostate cancer (Urine)	GC-MS	2,5-dimethylbenzaldehyde, 3-phenylpropionaldehyde, 4-methylhexan-3-one, dihydroedulan IA, hexanal, and methylglyoxal	Lima et al. [86]
Bladder cancer (Urine)	GC×GC TOF-MS	butyrolactone, 2-methoxyphenol,3-methoxy-5-methylphenol, 1-(2,6,6-trimethylcyclohexa-1,3-dien-1-yl)-2-buten-1-one, nootkatone, and 1-(2,6,6-trimethyl-1-cyclohexenyl)-2-buten-1-one	Ligor et al. [82]
Bladder cancer (Urine)	GC-MS	nonanal, 2-ethylhexan-1-ol, 1,1,4a-trimethyl-4,5,6,7-tetrahydro-3H-naphthalen-2-one, 5-ethyl-3-methyloxolan-2-one, phenol, 4-methylpent-3-enoic acid, 2-methoxyphenol, 3-methylheptan-2-one, 1,2,4,5-tetramethylbenzene, and Heptan-2-one	Lett et al. [83]
Bladder cancer (Urine)	HS-SPME-GM-M	2-butanone and 4-heptanone	Pinto et al. [84]
Colorectal cancer (Breath/Feces)	TD-GC-MS	Heptanoic acid; Acetone; 2,6,10-trimethyldodecane; n-hexane; Skatole; Dimethyl trisulfide	Śmiełowska et al. [53]
17 Diseases (Breath)	GC-MS	2-ethylhexanol; 3-methylhexane; 5-ethyl-3-methyloctane; Acetone; Ethanol; Ethyl acetate; Ethylbenzene; Isononane; Isoprene; Nonanal; Styrene; Toluene; Undecane	Nakhleh et al. [89]
Pancreatic ductal adenocarcinoma (Urine)	GC-MS	2-pentanone; Hexanal; 3-hexanone; *p*-cymene	Wen et al. [87]
Esophagogastric cancer (Breath)	SIFT-MS	Butyric acid; Hexanoic acid, Butanal; Decanal	Markar et al. (2018) [88]

**Table 2:** Comparative VOC extraction and detection methods.

Extraction Techniques			
	Advantages	Disadvantages	Limit of Detection (LOD)
SOLVENT EXTRACTION			
Liquid–Liquid (LLE)	Efficient polarity separation; selective industrial use	Limited solvent recovery time-consuming, loss of volume	ng/L to μg/L
Solid–Liquid Phase (SLE)	Effective solubility separation, simple process, solvent flexibility, high volume extraction	Large volume consumption, low extraction efficiency, heat sensitivity	ng/L to μg/L
Supercritical Fluid (SFE)	Eco-friendly, high-throughput, non-toxic, minimal waste generation	High initial cost, complex process, limited solvent selection	ng/L to μg/L
GAS EXTRACTION			
Thermal Desorption (TD)	No solvent use, high sensitivity, minimal sample preparation, no cross-contamination	Limited to semi-volatile and volatile compounds, costly	ppt to sub-ppb
Purge and Trap (PT)	Semi-volatile and volatile compounds, no solvent use, high sensitivity	Costly, complex setup, sample size limitations	ppt to ppb
Head Space (HS)	Minimal sample preparation, non-destructive, high sensitivity	Limited to semi-volatile and volatile compounds, matrix effect, calibration challenges	ppt to ppb
SORBENT EXTRACTION			
Solid Phase Microextraction (SPME)	Limited solvent use, minimal sample volume, high sensitivity	Limited fiber capacity, potential contamination, sample/fiber compatibility	ppt to ppb
Stir Bar Sorptive Extraction (SBSE)	High sensitivity, High extraction capacity, Solvent-free extraction	Loss of analyte, long extraction time, coating degradation	ppt to ppb
Solid Phase (SPE)	Cost-effective, improved reproducibility and sensitivity, high recovery rates	Limited to liquid samples, matrix effect, time consuming	sub-ppb to low ppb
Needle-Based (NBE)			ppt to sub-ppb
**Detection Methods**			
	**Advantages**	**Disadvantages**	**Limit of Detection (LOD)**
Gas Chromatography (GC)	High sensitivity, excellent separation power, high resolution, rapid analysis	Limited to gaseous and volatile compounds, detector limitations	ppt to sub-ppb
Liquid Chromatography (LC)	Polar and nonpolar volatiles, higher sensitivity, quantitative analysis	Higher cost and maintenance, solvent interferences, complex method development	low ppb to sub-ppb
Nuclear Magnetic Resonance (NMR)	High sensitivity, non-destructive, versatility, high reproducibility	Time-consuming, limited throughput, complex data interpretation	1–10 mM and 10–500 μM
Mass Spectrometry (MS)	High sensitivity, versatility, quantitative analysis, non-destructive	Expensive maintenance, sample preparation, matrix effect	ng/mL to fg/mL

**Table 3:** Examples of analytical methods for VOCs.

Sample Matrix	Analytical Methods	Detected VOCs	References
Urine	GC-SRI-TOF-MS and HS-SPME	16 VOCs: Acetone; 2-butanon; 3-methyl-2-butanone; 2-pentanone; 3-methyl-2-pentanone; 4-methyl-2-pentanone; 2-hexanone; 3-hexanone; 2-heptanone; 4-heptanone; Dimethyl sulfide; Allyl methyl sulfide; Methyl propyl sulfide; Furan; 2-methylfuran; 3-methylfuran	Mochalski et al. [134]
Urine	HS-SPME/GC-MS	21 VOCs	Monteiro et al. [122]
Urine	NTME/GC-MS	103 VOCs	Porto-Figueira et al. [118]
*Staphylococcus aureus*	SPME and SBSE	12 VOCs: Acetaldehyde; Ethanol; 1-methyl-1-propylhydrazine(methyltrisulfanyl)methane; 3-ethyl-2,5-dimethylpyrazine; Acetic acid; Formic acid; Benzaldehyde; 2-hydroxybenzaldehyde; Acetamide; 1,3,5,7-Tetraazatricyclo[3.3.1.3.7]decane; 4-methylquinoline; Isoquinoline-1-carbonitrile; quinoline-4-carbaldehyde; 1H-indole	Berrou et al. [114]
Urine	SPME-LC-MS	22 VOCs	Luczykowski et al. [107]

## Data Availability

No new data were created or analyzed in this study.
